# Analyzing hospital behavior across different payment mechanism as a response to financial incentives – a scoping review

**DOI:** 10.1186/s12913-026-15029-5

**Published:** 2026-07-02

**Authors:** Barbara Więckowska, Katarzyna Byszek, Katarzyna Luchowska, Agnieszka Głąb, Anna Marszałek, Melanie Raczek-Żeromska, Monika Raulinajtys-Grzybek

**Affiliations:** 1 Healthcare Innovation Unit, Institute of Social Economy, Collegium of Socio- Economics, SGH Warsaw School of Economics, al. Niepodległości 162, Warsaw, 02-554 Poland; 2 Management Accounting Department, Collegium of Business Administration, SGH Warsaw School of Economics, Warsaw, Poland

**Keywords:** Financial incentives, Hospital care, Hospital behavior, Literature review

## Abstract

**Background:**

This scoping review maps published evidence on financial incentives linked to hospital payment mechanisms and on the hospital-behavior endpoint categories examined in the literature. It provides an evidence map for subsequent systematic review work within the FLASH project, rather than estimating the direction or magnitude of effects.

**Methods:**

The review was reported in accordance with PRISMA guidelines. Authors searched PubMed and Cochrane for English-language records published between January 2013 to February 2023. Data was independently screened and extracted by two authors. In total, 140 full-text records were included and classified country, publication year, payment mechanism, financial-incentive category, and endpoint category.

**Results:**

Since 2015, the number of publications on financial incentives in healthcare has generally increased. Most included publications covered changes in the United States, Taiwan, China, and the United Kingdom. The identified incentives were mapped within payment-mechanism categories, including Activity-Based Payment, Incremental, Consolidated, and Budget mechanisms, following WHO/OECD and Urban Institute classifications. Activity-Based Payment was the most frequently studied category; the endpoint categories most often examined in this group were efficiency of spending funds (42 publications), access (27 publications), and hospitals' finances (22 publications). Studies of incremental mechanisms, mainly Pay-for-Performance (P4P), most often examined quality (41 publications) and efficiency of spending funds (24 publications), while consolidated mechanisms were represented less frequently across endpoint categories. These counts describe the distribution of the literature and do not indicate effect direction, magnitude, or statistical significance.

**Conclusions:**

The review identifies where evidence on hospital payment mechanisms, financial incentives, and hospital-behavior endpoint categories is concentrated and where gaps remain. The most researched endpoint categories are linked to efficiency of spending funds, quality, access, and hospital finances. Payer spending and budget-based mechanisms are comparatively less represented. We identify priorities for subsequent systematic review and for a database that extracts the direction, magnitude, and context of reported effects.

**Supplementary Information:**

The online version contains supplementary material available at https://doi.org/10.1186/s12913-026-15029-5.

## Introduction

Healthcare systems around the world face an everlasting challenge: delivering high-quality care while managing escalating costs [1]. In pursuit of this goal, a multitude of hospital payment mechanisms have been introduced [2]. These mechanisms incorporate specific financial incentives (gains or losses [3]) intended to stimulate hospital behavior [4], in order to reach desirable outcome such as healthcare utilization, efficiency, and quality of care [2].

Previous reviews have highlighted considerable heterogeneity in hospital payment mechanisms. For example, an overview of thirty-four Pay-for-Performance (P4P) programs across OECD countries showed substantial variation in program design and implementation context [5]. This heterogeneity complicates efforts to synthesize how financial incentives are embedded in payment mechanisms and which healthcare provider (hospital)-behavior endpoints are studied.

Healthcare financial systems comprise various hospital payment mechanisms (even for one type of healthcare providers) through which third party payers implement financial incentives aimed at achieving specific desired outcomes. Understanding what financial incentives are linked to which hospital payment mechanisms and how they are linked is a necessary first step before evaluating whether there is a possibility to reach more efficient healthcare delivery. In other words: financial incentives do not arise from hospital payment mechanisms per se, but from the specific features built in these mechanisms and changes in those features.

Previous systematic reviews have often been limited to specific countries, payment mechanisms, clinical areas, or endpoint categories. To date, eight systematic reviews [5–12] have analyzed the association between financial incentives, hospital payment mechanisms and hospital behavior; however, some questions remain unanswered. First, two of the reviews only focuses on one country system (the UK, Germany) [6, 9]. Four reviews regard only one model (P4P or VBHC) [5, 7–9]. Two reviews are limited to one health area (stroke or orthopedic surgery) [11, 12]. One review focuses exclusively on quality of care endpoints [10]. Our scoping review fills gaps in literature by considering a broad range of endpoints related to hospital-behavior motivated by various incentives associated with specific payment mechanisms. The primary aim is to identify and map published evidence on financial incentives used in hospital payment mechanisms and the endpoint categories examined in that evidence. Specifically, we map: (1) research on changes (financial incentives) in hospital payment mechanisms; (2) endpoint categories used to assess hospital response for financial incentives; and (3) the distribution of publications by hospital payment mechanisms and endpoint categories. By identifying and classifying the relevant literature in this article, we provide a base for further full systematic review focused on how specific financial incentives affect different endpoint categories.

This review is a part of the FLASH Project – Flexible Approaches to Support Health through financing. FLASH is a Horizon Europe research project (grant agreement no. 101095424) that examines healthcare-financing mechanisms in Europe. The current scoping review contributes to FLASH by mapping how hospital payment mechanisms and financial incentives have been studied internationally, providing a foundation for subsequent work on financing models, hospital incentives, resilience, efficiency, and equity.

## Methods

This study is part of a broader research project examining the role of financial incentives in shaping hospital behavior across various financing mechanisms. To achieve the project’s objectives in a methodologically efficient and coherent manner, the literature review was conducted using two complementary approaches to evidence synthesis: a scoping review and a systematic review. The specific goal of the scoping review was to identify research on financial incentives implemented to hospital payment mechanisms and to classify them by the endpoint category. A mapping function was deemed necessary for further attempts at estimating the effect, given the heterogeneity and conceptual inconsistency observed in the literature. The systematic review (presented separately) was designed to utilize the evidence map developed as part of the scoping project to classify the types of financial incentives, estimate the direction and magnitude of the influence of financial incentives on hospital behavior.

We performed the overall search in a systematic way to minimize the potential bias. Specifically, the PRISMA-P (Preferred Reporting Items for Systematic Reviews and Meta-Analyses) guidelines were followed to design the search strategy [13] and PRISMA extension for scoping review (PRISMA-ScR) was used as a guide to report the findings [14].

### Data sources and search strategy

Given the common themes of both reviews (scoping and systematic), a single search strategy was developed and applied. This approach ensured terminological consistency, avoided duplication, and ensured that the evidence base for the systematic review was embedded within the broader framework of the scoping review.

In the first stage of searching for scientific reports, we reviewed two electronic medical information databases: PubMed and Cochrane. The search strategy was developed by the research team (AG, BW, KB, MRG, AM).

The search strategy was based on the PIO model [15–17], the elements of which were interpreted broadly for the purposes of the scoping review, in line with the exploratory nature of this approach.

To build search terms for the identification of financial and organizational aspects affecting hospital providers, we identified keywords related to the population, intervention, and outcomes of interest. We also considered different word forms, synonyms, and subject heading terms appropriate for each database. Boolean operators and Medical Subject Headings (MeSH) were used to optimize search results. The exact keywords for each database are provided in Supplementary Annex 1. The search was limited to English-language literature published between January 2013 and February 2023 (the last 10 years). This 10-year window was chosen to capture contemporary payment and contracting arrangements after major post-2010 reforms and the growing adoption of value-based payment approaches, including the Affordable Care Act in the United States [18] and bundled-payment strategies [19]. Earlier incentives often operated in different regulatory, contracting, clinical, and cost-accounting environments, making them less comparable with current practice. The end date reflects the date of the final database search.

### Eligibility criteria

Studies were eligible if their focus was on payment mechanisms in hospital care. Both qualitative and quantitative studies were included in the study. All primary epidemiological observational study designs (i.e., cross-sectional, cohort, case-control studies), ecological studies, and experimental studies were eligible. Table 1 presents all inclusion and exclusion criteria.

**Table 1:** Inclusion and exclusion criteria

	Inclusion	Exclusion
Population	hospital, inpatient providers	outpatients, primary care providers; countries in Africa and South America
Intervention	financial incentive implemented to hospital payment mechanism	no intervention, overview of the health system, intervention not related to hospital payment mechanism
Endpoints	any change in the behavior of healthcare providers resulting in e.g. changes in the quality of treatment, reduced queues, larger number of patients	
Other	only in English, since 2013	opinions, commentaries, editorials, and studies without access to full-text publications, systematic review

Source: Authors’ work

#### The process of selecting and classifying data

After searching the databases, we removed duplicates in the found articles, and the found systematic reviews were additionally searched for studies included in the analysis.

The study selection process included two stages:

- Stage 1: Title and Abstract Screening:

In the first stage (verification of titles and abstracts), the selection was made independently by two analysts (BW, KB, AG, AM, MRG). At this level, all reports deemed useful by at least one of the analysts were included in the next stage.

- Stage 2: Full-Text Review:

In the next stage (verification of full texts) the selection was made independently by two analysts (BW, KB, AM, MRG, KL, MRŻ). At this stage, the pair of analysts independently made the decision to ultimately include the study in the analysis. Any disagreement about the eligibility of studies was resolved through discussion and consensus among all co-authors, as recommended in the literature [13]. In case of discrepancies during the verification of the studies, the final position was established by consensus, or the assistance of a third analyst was used based on the full texts of the reports.

### Data extraction and quality assessment

A data extraction form was developed and pre-tested on first 10 full-text publications (BW, KB, AG, AM, MRG). The form was iteratively adjusted to extract all relevant information. Standardized tables were created a priori to extract the following data: author, year of publication, country of the trial, the study objective, study design type, period of the trial, population, intervention/incentives, category of the financial mechanism, the assessed final effect related to the financial mechanism and other important information regarding the financial mechanism.

### Data collection and analysis

We used standard methodological procedures and performed a structured descriptive synthesis. We identified the categories (themes) relevant to the review objective. First, the study classified hospital payment mechanisms using OECD/WHO classification [20] and the Urban Institute classification [21] showed in the table below (Table 2).

**Table 2:** Classification of hospital payment mechanisms

Main category	Hospital payment mechanism	Description	
Activity- Based Payment	FFS^5^	Fixed payment for each unit of service without regard to outcomes. It is typically paid retrospectively by billing for each service or patient contact.	
	Per diem^5^	Fixed amount per day for inpatient stay, which may vary by department, patient, clinical characteristics, or other factors.	
	DRG^5^	Payment paid to hospitals per admission or discharge, whereby patients are classified into groups (DRGs) based on diagnosis and procedures.	
Budget	Global budget^5^	A prospective lump-sum payment to a healthcare provider to cover aggregate costs over a specific period for a set of services independent of the actual volume provided.	
Consolidated	Bundled payment^5^	A single payment covering a bundle of distinct goods and services required for the treatment of a given medical condition based on clinical practice guidelines.	
	Capitation^5^	Prospective fixed lump-sum payment per person enrolled for care with a provider within a given period (typically one year) covering a defined set of services, independent of whether the services are provided.	
Incremental	P4P^5^	Payments to health care providers for meeting specific performance targets, such as process quality or efficiency measures, or penalties for poor outcomes, such as medical errors or avoidable readmissions.	
	Shared savings^6^	A form of payment in which a provider or a provider organization shares generated savings with the payer when actual spending for a defined population is less than a target amount. Under shared savings—also referred to as one-sided or upside-only—the recipient is not at risk for overspending.	

Source: OECD 2019, Urban 2016

Different categories of endpoints were identified in our scoping review, including measures related to access, quality, hospitals’ finances, and payers’ spending. The classification of endpoint categories and measures was developed from the perspective of healthcare financing. The full classification is presented in the table below (Table 3).

**Table 3:** Classification of the endpoint categories

Endpoint categories	Endpoint measures
Access	Case mix (patients/services) Number of patients Number of services Waiting time
Quality	Survival/mortality/life expectancy Indicators of clinical quality of treatment Cure/prevention Adverse events Patient satisfaction
Hospitals’ finances	Upcoding Revenues Costs Financial risk
Efficiency of spending funds	LOS Readmission Patient transfer
Payer	Spending

Source: Authors’ work

The extracted data was analyzed descriptively, consistent with the objectives of the scoping review. Results were presented using tabular and graphical data presentations, with particular emphasis on frequency distributions and bar graphs to illustrate the number and characteristics of the included evidence. This approach allows for a structured mapping of identified studies by publication year, country (defined as the geographic setting examined within the study), hospital payment mechanism and endpoint category. In accordance with the scoping review methodology, the publications were not subjected to a formal quality assessment of the included studies, and no statistical analyses were performed.

### Text analysis with MAXQDA

The qualitative team (BW, KB, AM, MRG, KL, MŻR) initiated the process by transferring full text articles into MAXQDA (MAXQDA Analytics Pro 2022 Version) for efficient data organization. MAXQDA software was subsequently employed for both first- and second-level coding and analysis. Rigor and validity were meticulously addressed during the data cleaning and organization phase, with the qualitative team overseeing the review of 100% of abstracted articles. Following the coding phase, another researcher scrutinized 100% of the coded text in MAXQDA to evaluate the validity of the coding scheme.

In instances of disagreement in either abstracted or coded text, the team engaged in group discussions during their weekly meetings, ultimately reaching a consensus. This thorough over-reading process served to affirm the validity of their interpretations. Once the data underwent independent coding, over-reading, and group discussions among the three qualitative researchers, a thematic synthesis was conducted. This synthesis involved identifying and grouping related codes and categories linked to financing mechanisms and endpoint categories. The creation and identification of codes and themes unfolded in an iterative fashion.

To ensure the rigor and validity of their findings, the team independently coded and developed themes. Subsequently, the team engaged in discussions about theme development and identification until a consensus was reached. This rigorous process underscored the robustness of the qualitative analysis and thematic synthesis in their research.

## Results

### General characteristics of included studies

**Fig. 1:**
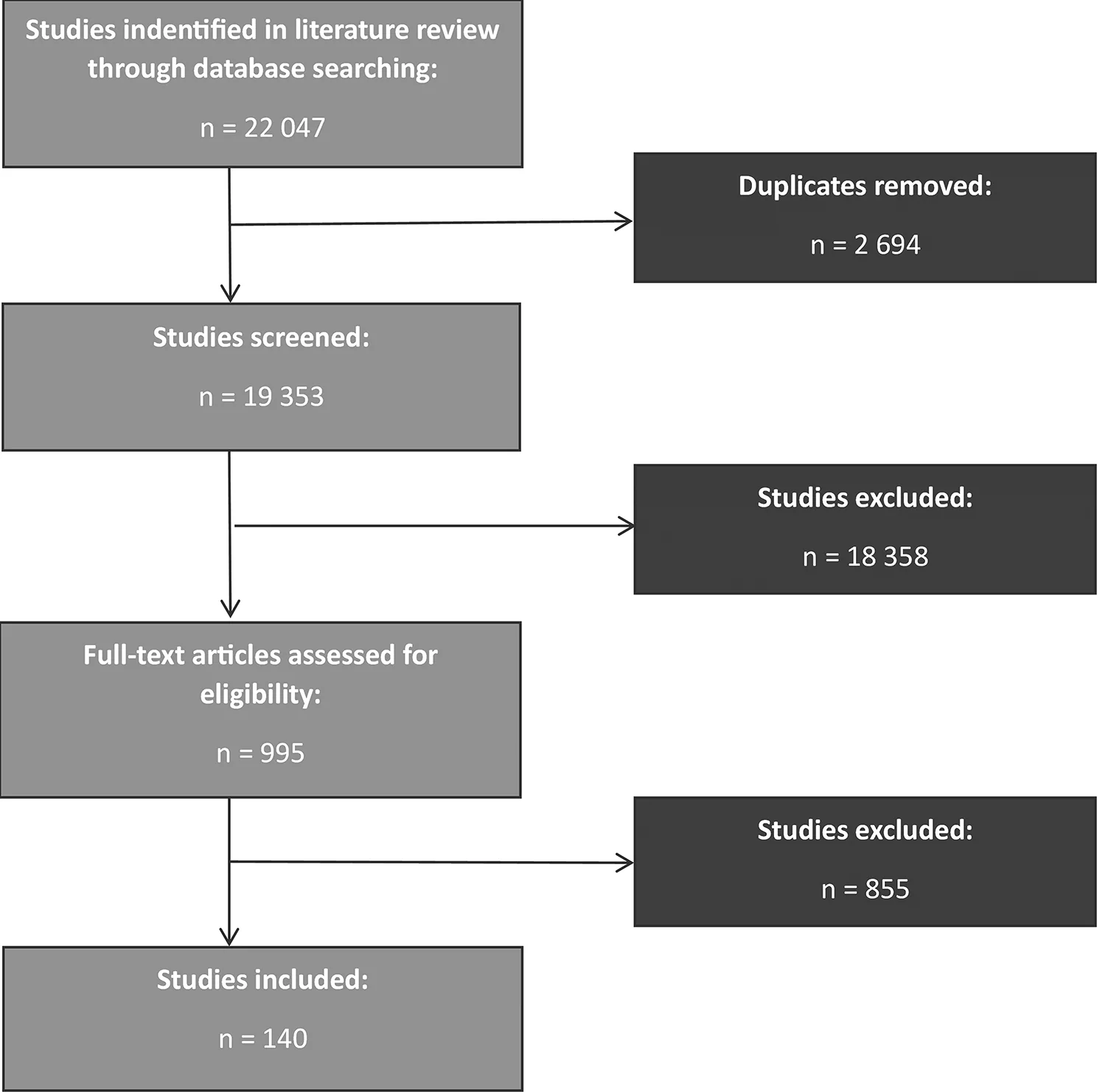
PRISMA flow diagram of study selection. Source: Authors’ work

The database searches yielded 22,047 publications. After removing duplicates and reviewing the titles and abstracts, 995 publications were included in the screening based on full text. The number of excluded full-text articles with reasons is presented in Fig. 1. A hierarchy of article exclusion was used: first, articles related to the type of publication, i.e., opinions, without full access, with languages other than English, and systematic reviews, were rejected. The population, intervention, and endpoints were then assessed. This procedure allowed each article to be assigned one reason for exclusion from the analysis. Ultimately, 140 records on financial aspects were included in this review. The stages of the selection process are showed below.

**Fig. 2:**
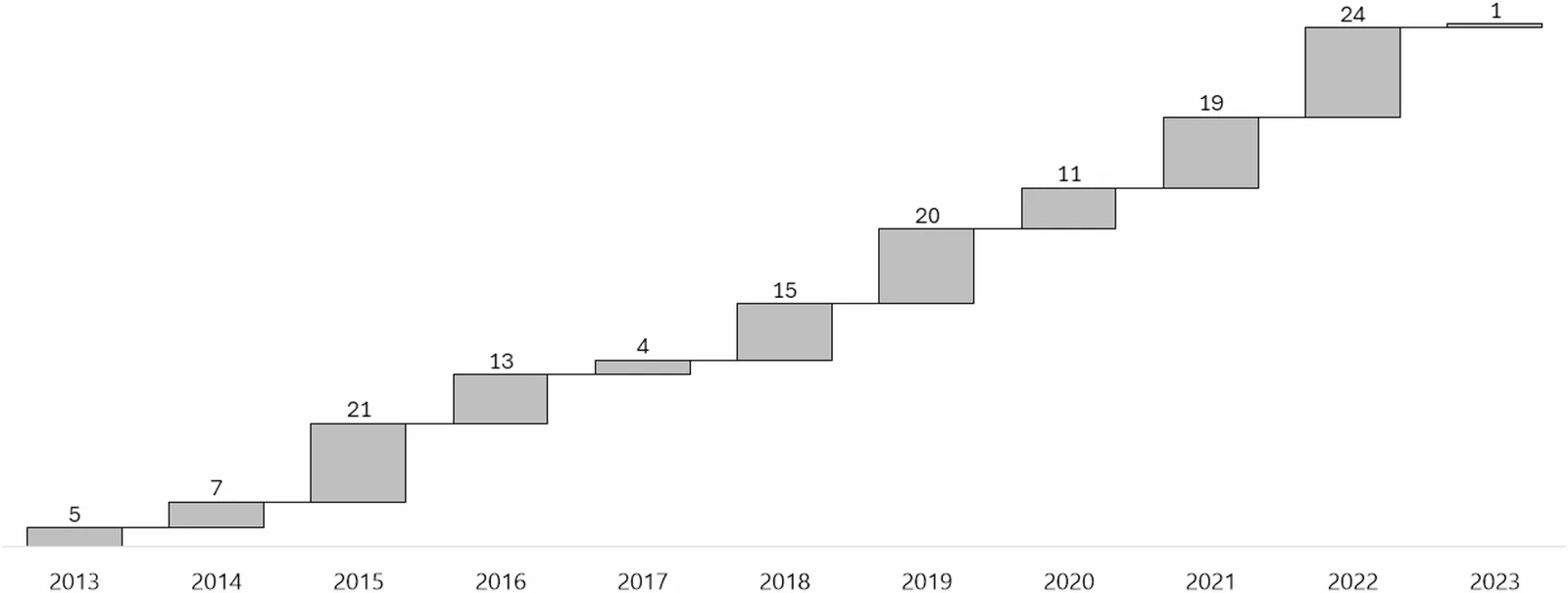
Number of publications by year. Source: Authors’ work

Figure 2 presents the number of studies regarding incentives influencing health care providers, published from January 2013 until February 2023. As observed in Fig. 2, this topic is of continuing interest to researchers, which is reflected in the increasing number of publications. The largest number of publications (from 140 publications) appeared in 2022 (17%), 2015 (15%) and 2019 (14%). The smallest number of publications in the analyzed period have been published in 2013 (4%), 2017 (3%) and 2023 (1%). The small number of publications from 2023 is expected because the search was conducted in February 2023, when many studies from that year had not yet been published or indexed.

**Fig. 3:**
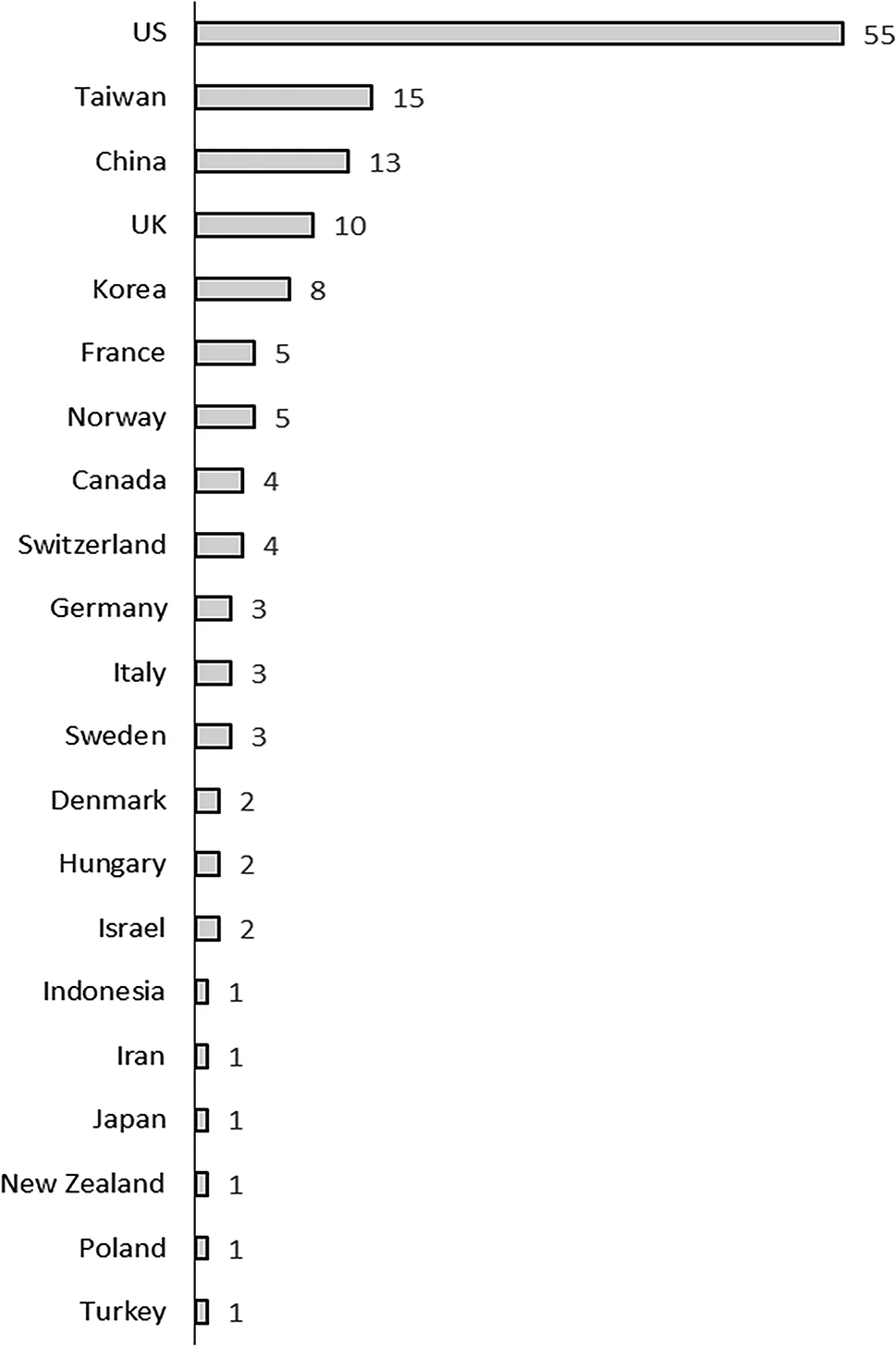
The number of publications by country. Source: Authors’ work

Figure 3 presents the number of publications by country. The publications found covered twenty-four different countries. The largest number of publications relate to the United States (39%), Taiwan (11%) China (9%), the United Kingdom (7%) and South Korea (6%). The share of each remaining country did not exceed 4% of the included publications.

### Payment mechanisms

Table 4 shows the number of publications by hospital payment mechanism described in the articles. The largest number of publications is focused on the Activity-Based Payment main category (43%), and among them, the largest number of publications is on DRG-based payment (79%). The second largest number of publications is related to the Incremental main category (40%), with the largest number of publications dealing with the Pay-For-Performance mechanism (98%). In third place is the Consolidated main category (11%), which corresponds mostly to Bundled Payment financing (94%). Budget-based mechanisms were least frequently represented (5%).

**Table 4:** Mechanisms described in the articles (number of articles)

Category of payment mechanism	Payment mechanism
Budget (7)	Global Budget (7)
Activity-Based Payment (61)	Fee-For-Service (9)
	Payment Per Diem (3)
	DRG (48)
	Case Payment (1)
Consolidated (16)	Bundled Payment (15)
	Capitation (1)
Incremental (56)	Pay-For-Performance (55)
	Shared savings (1)

Source: Authors’ work

**Fig. 4:**
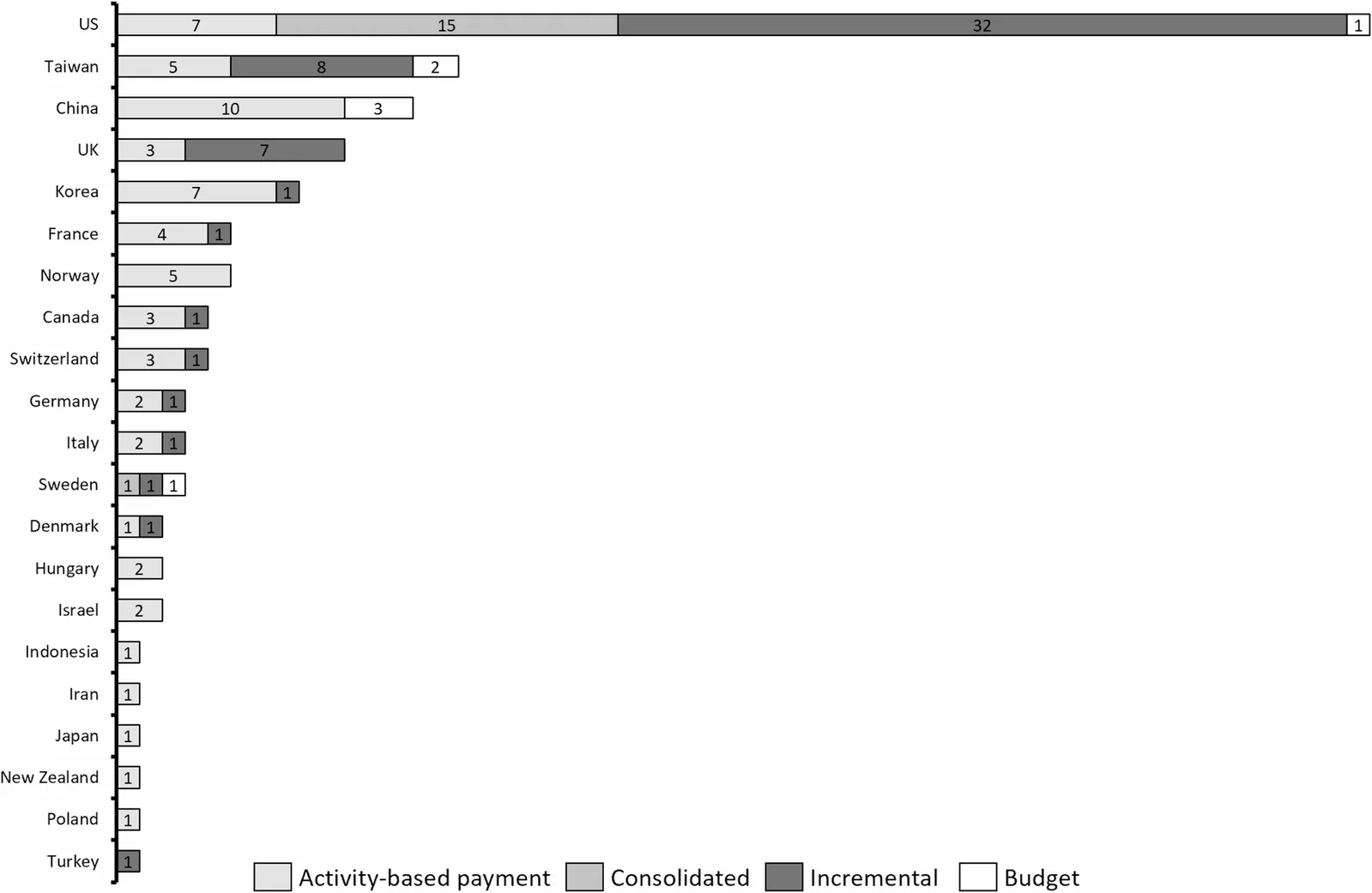
Number of publications with details on financing mechanisms by country. Source: Authors’ work

Figure 4 illustrates the number of publications on each country, organized by financing mechanism categories. Publications on Turkey, Poland, New Zealand, Japan, Israel, Iran, Indonesia, Norway, and Hungary focused on only one category of health financing mechanism, with publications on Incremental in Turkey, and Activity-Based Payment in the remaining countries. Publications from Denmark, Sweden, Italy, Germany, France, Switzerland, China, and Canada covered two different categories of financing mechanisms. In China, publications focused on Activity-Based Payment and Budget, while in Denmark, Italy, Germany, France, Switzerland, and Canada, the focus was on Activity-Based Payment and Incremental mechanisms. Publications from Taiwan and Sweden examined three categories. In Taiwan, the focus was on Activity-Based Payment, Incremental, and Budget, while in Sweden, it spanned Incremental, Consolidated, and Budget categories. Publications from the United Kingdom focused on two categories: Activity-Based Payment and Incremental. Research from the United States encompassed all four major categories, with the most studies (58%) focusing on the Incremental category and the smallest share (2%) on the Budget category. Publications on Consolidated mechanisms accounted for 27%, and Activity-Based Payment for 13%.

### Endpoints categories

As Table 5 shows, the most common endpoint category discussed in the articles was efficiency of spending funds (73 publications; 30%). Within this category, studies discussed the impact of financial incentives on the length of stay (38; 52% of this category) and on readmissions (32; 44%). The next categories had a comparable frequency in publications - Quality (64, 26%), Access (50; 20%) and Hospitals’ finances (40; 16%). In publications examining quality, the most frequently examined were indicators of clinical quality of treatment (23, 36%) and survival/life expectancy (18, 28%). In the Access category, the most common studies were those assessing the impact of financial incentives on the number of services (27, 54%) and case mix (15, 30%). The cost of healthcare was the dominant subcategory within hospitals’ finances (21, 53%). Payer spending was the least common category (20 publications in total, 8%).

**Table 5:** Endpoint categories described in the articles

Endpoint category	Endpoint measures
Access (50)	Case mix (patient/services) (15)
	Number of patients (4)
	Number of services (27)
	Waiting time (4)
Quality (64)	Survival/life expectancy (18)
	Indicators of clinical quality of treatment (23)
	Cure/Prevention (4)
	Adverse events (14)
	Patient satisfaction (5)
Hospitals’ finances (40)	Upcoding (10)
	Revenues (8)
	Costs (21)
	Financial risk (1)
Efficiency of spending funds (73)	LOS (Length of stay) (38)
	Readmission (32)
	Patient transfer (2)
Payer (20)	Spending (20)

Source: Authors’ work

**Fig. 5:**
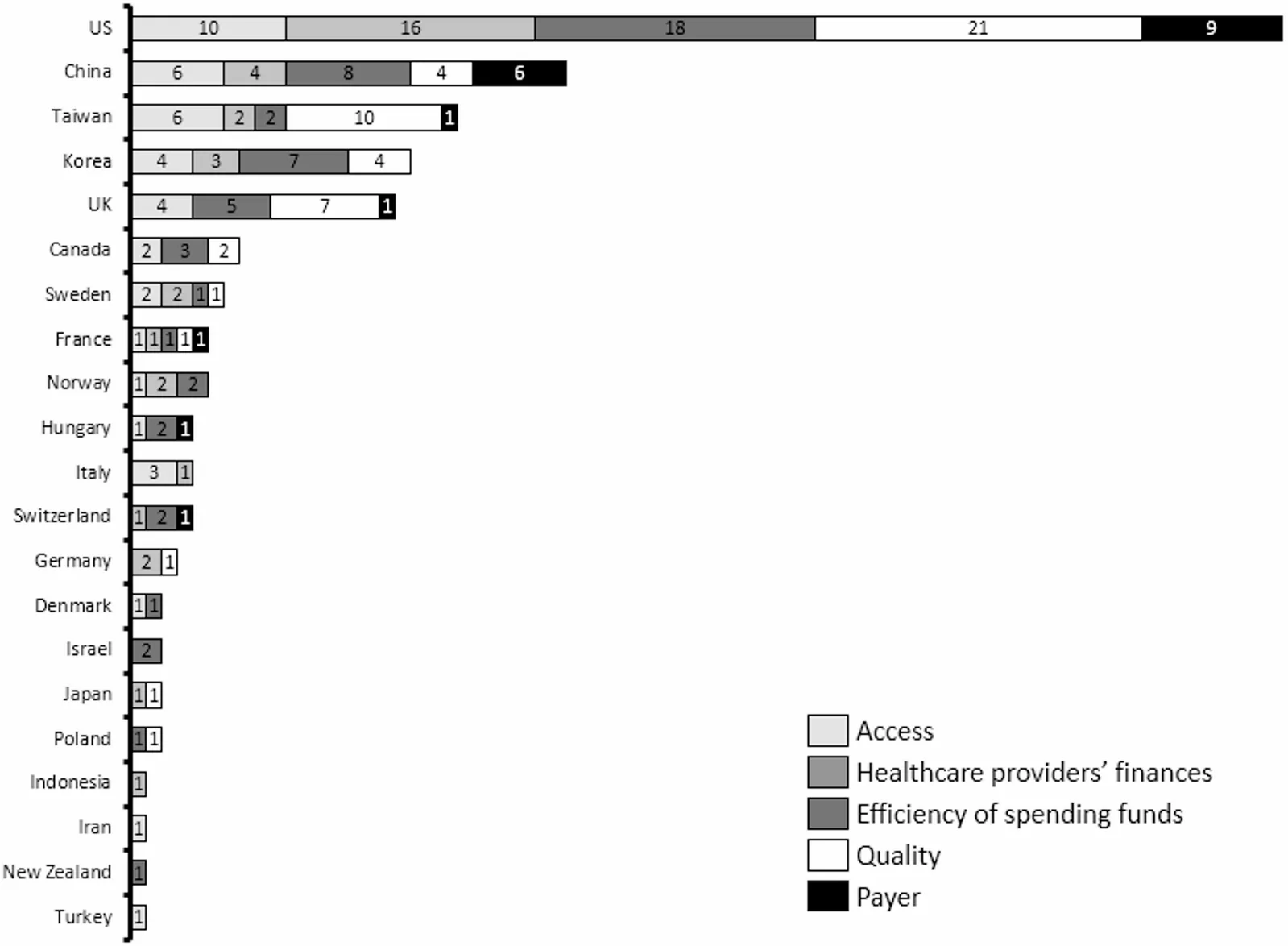
Number of publications with details on endpoints by country

Figure 5 presents a comparative analysis of the volume of publications on each country across five key endpoint categories: Access, Hospitals’ Finances, Efficiency of Spending Funds, Quality, and Payer spending. Some publications covered more than one endpoint category. The United States accounted for the highest number of publications, with 55 publications covering 74 endpoint-category mentions across five categories. China, Taiwan, South Korea, and the United Kingdom followed, with contributions in categories such as Efficiency of Spending Funds and Access. Multiple endpoint categories (three or more) were explored in publications on the United States, Taiwan, China, the United Kingdom, South Korea, Sweden, Norway, Hungary, Canada, France, and Switzerland. This pattern indicates broader coverage of endpoint categories in publications from these countries, rather than evidence of stronger or weaker effects. It may also reflect the unequal distribution of included studies across countries, as those most frequently represented in the review are more likely to include analyses of multiple endpoint categories. Publications from Canada, Sweden and France showed a more balanced but lower output. In Italy, two categories were examined: access and hospitals’ finances. Publications from Denmark focused on access and efficiency of spending funds; from Germany – on hospitals’ finances and efficiency of spending funds; from Japan – on hospitals’ finances and quality; and from Poland - access and efficiency of spending funds.

Single entries from Indonesia and New Zealand were captured. The distribution highlights significant disparities in the geographic concentration of research on payment mechanisms and financial incentives.

### Results by publication date

**Fig. 6:**
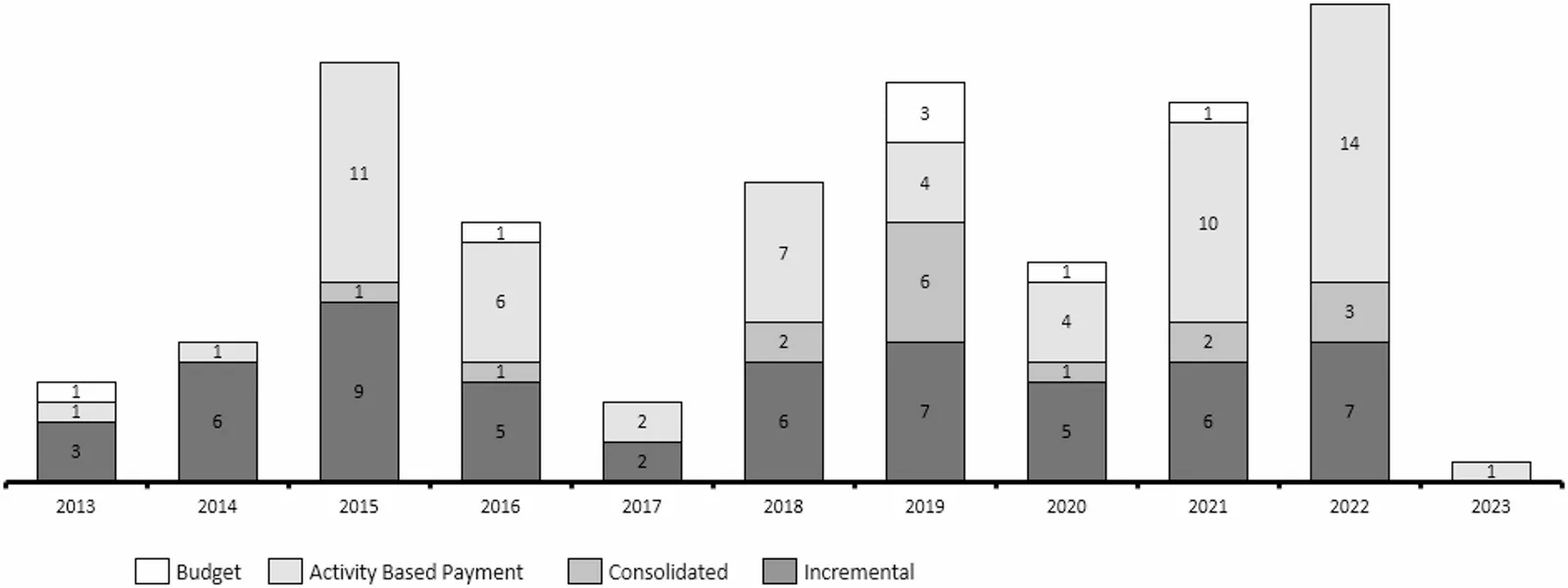
Number of articles by hospital payment mechanism and publication date. Source: Authors’ work

**Fig. 7:**
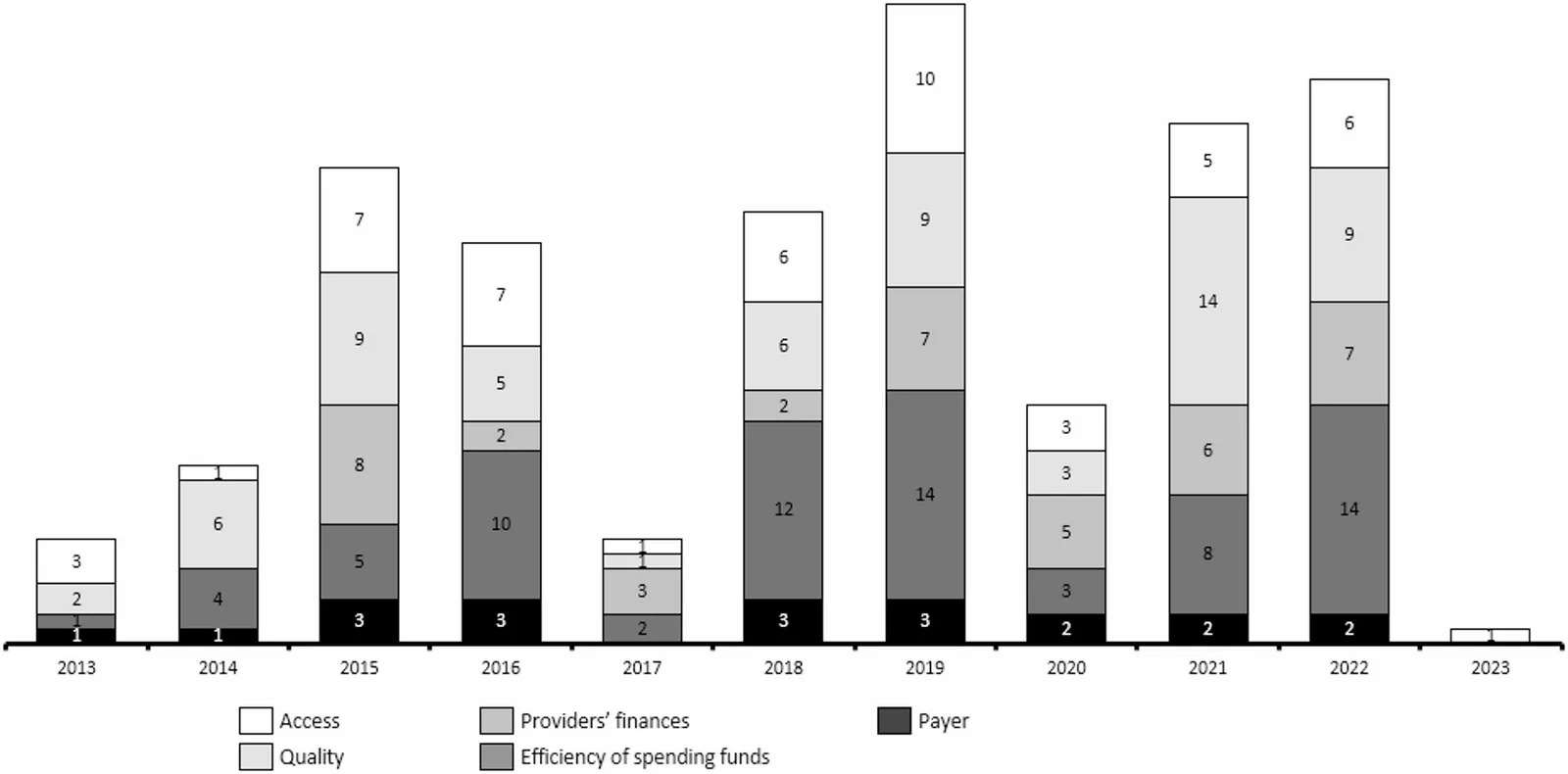
Number of articles by endpoints and publication date. Source: Authors’ work

When examining the distribution of publications over time, attention should be paid to the cyclically increasing total number of publications, which may indicate a recurring interest in the topic of financing mechanisms and their impact on the functioning of healthcare. At the same time, there are no visible changes over time in terms of the structure of the hospital payment mechanisms discussed, as well as in the areas studied of the endpoints (Figs. 6 and 7).

### Mapping payment mechanisms with endpoint categories

Table 6 summarizes the endpoint categories by hospital payment mechanisms. The largest number of studies was related to the impact of Activity-Based Payment on different aspects of hospital performance. The most frequently studied effect was the change in efficiency of spending funds (42 publications), then on access to healthcare (27 publications), hospitals’ finances (22 publications), and slightly less frequently on quality and payer. In the case of the impact of incremental mechanisms (and Pay-for-Performance, the most common in this category), it was most often related to quality (41 publications), then to efficiency of spending funds (24 publications). Consolidated mechanisms were examined less frequently, and all aspects were analyzed proportionally. In the case of the budget, the analysis of the relationship concerned mainly access and hospitals’ finances.

**Table 6:** Endpoint categories by payment mechanisms

Category of payment mechanism \ Endpoint category	Access	Efficiency of spending funds	Hospital’ finances	Payer	Quality
Activity Based Payment	27	42	22	8	15
Budget	7	0	3	2	2
Consolidated	5	7	5	8	6
Incremental	11	24	10	6	41

Source: Authors’ work

### Limitations

Although this scoping review presents structured evidence map, several methodological and contextual limitations should be considered.

Time Restriction: The search was limited to studies published from January 2013 to February 2023. Although this period was selected to focus on contemporary payment and contracting systems, it may have excluded earlier foundational research on healthcare financing and hospital behaviour.

Geographical Bias: A significant portion of the identified studies covered the United States, United Kingdom, and Australia, suggesting a geographical bias. This may limit the generalizability of the findings to other regions, particularly low- and middle-income countries where healthcare systems and payment mechanisms differ substantially. Additionally, the results may underrepresent evidence from other regions and non-English sources.

Publication Bias: The review included only published studies, which may introduce a publication bias. Unpublished or grey literature, including reports from governments or international organizations, may contain valuable insights but were not considered in this study.

Potential for Reviewer Bias: Although two authors independently screened and extracted data, the classification of complex payment mechanisms and endpoint categories required interpretation. Disagreements were resolved through consensus to reduce this risk. There were no external reviewers to possibly guard against the reviewer-researcher bias, however, the approach was discussed and approved during the consortium meeting.

## Discussion

This scoping review identifies and maps literature on financial incentives implemented in hospital payment mechanisms. In includes 140 publications. The mapping included a broad range of endpoints across multiple provider payment mechanisms, whereas prior research was limited to a single country [6, 8], a single payment mechanism [5, 7, 9, 10], a single clinical area [11, 12], or a single endpoint category [6].

The growing number of publications over the years may be related to globally introduced programs, such as the Affordable Care Act (ACA), which expanded pay-for-performance (P4P) strategies in the United States [22]. The impact of this legislation has been analyzed in the years following its implementation, potentially explaining the increase in the number of articles around 2015.

The analysis of the payment mechanisms shows the prevalence of activity-based payment (43% of all, mostly DRG) and incremental mechanisms (40%, almost entirely P4P). The high frequency of DRG-related publications can be linked to the international diffusion of case mix systems after their initial implementation introduction in 1980 in the US [23]. Globally, DRG-based systems are among the most used financial mechanisms in hospitals across the USA, Europe [24], and Asia, which may explain their prominence in the literature [25].

P4P attracts much research attention compared to its actual impact on provider’s revenue. This mechanism, due to its cause-and-effect design, enables the evaluation of the impact on specified indicators, and therefore, is of interest of the researchers. The implementation of pay-for-performance (P4P) models became more widespread around 2010. Conducted research can concentrate on differences between different P4P programmes’ design and implementation [5], or on their divergent effects on process-of-care and patient outcomes [7]. While P4P is often classified as a distinct payment mechanism, it typically serves as an enhancement to existing payment models and can be evaluated within various financing frameworks.

There is an underrepresentation of budget and consolidated mechanisms in the database – only 5% and 11% of publications were related to that area. This is even though global budgets are still a significant mechanism used in many European systems, especially in hospital settings. European Observatory on Health Systems and Policies [26] mentions global budgets among the most common payment mechanisms for hospitals, next to per diems, and DRG-based case payments.

The geographical distribution of publications shows hight representation of studies from a small number of high-income settings, 39% of publications were from the US alone. The presence of multiple competing payers in the US creates a variety of payment mechanisms within a single country [27]. Many European countries are barely represented despite running interesting financing reforms. This may partly reflect our restriction to English-language publications; local studies published in national languages would not have been captured. In some of the European countries, there is one primary public payer, which may partly explain the lower diversity of payment mechanisms found in the analyzed articles. It is important to note that our review focused on articles in which payment mechanisms incorporated financial incentives and in which endpoint categories related to provider behavior were examined.

Classification of endpoints used in this study was developed from a healthcare financing perspective. Consequently, length of stay (LOS) and readmissions were classified under “Efficiency of spending funds,” as shorter hospital stays and lower readmission rates may indicate more efficient use of available financial resources. However, we acknowledge that such categorization may vary depending on the analytical perspective and healthcare context, as these outcomes are also commonly interpreted as indicators of quality [28] or access [29] in the literature.

Endpoints reveal researchers’ interest, which partly reflects what is included in the payment models worldwide. Efficiency of spending was mostly represented (30% of publications), driven by LOS and readmissions. Quality and access were the next most represented categories. Specifically in the access category, the most popular were very traditional measures, such as number of services or case mix. What is worth mentioning, patient-related endpoints, such as satisfaction, cure/prevention, or waiting time, are underrepresented. Financial risk perspective was also not visible in the database, which is interesting given the policy interest in risk-sharing mechanisms in healthcare [30].

Table 6 shows that the research predominantly links the mechanism with the outcome that was designed to be influenced by a change in financing hospital care. This pattern directly addresses our third and fourth research questions concerning whether specific outcome measures are linked to specific financing mechanisms.

Activity-based payment is examined mostly through the lens of efficiency of spending funds (42 publications) and access (27 publications). Those mechanisms, particularly DRGs, reward providers for every discharge, and provide financial incentives to shorten LOS, adjust case mix, and avoid readmissions. These endpoints have been prioritized in the research.

What is interesting, there is limited number of publications on quality endpoints in relations to activity-based payment. As activity-based mechanisms reward volume rather than outcome, they may have negative quality consequences [31]. This mechanism remains relatively underrepresented in the research, compared to the efficiency effects.

Incremental mechanisms (mainly P4P), on the contrary, are dominated with research on quality endpoint (41 publications), followed by efficiency (24 publications). This is rooted in the design of those schemes, which link payment to specific performance targets.

A scarcity of research on P4P with access or provider finances endpoints can draw attention. Negative impact in these areas could contradict the desired pro-quality effect. This is especially relevant given that some publications suggest P4P schemes may encourage patient selection [32, 33].

Consolidated mechanisms show a more even distribution across endpoint categories. Given their more hybrid nature and combination of features typical for different financial mechanisms, these results are justified.

What is most striking is the distribution of publication in the budget category. Firstly, as mentioned before, the overall number of publications is disproportionally low taken the practical use of budgets in healthcare financing. Additionally, the research is concentrated on the access endpoint, and to much lower extent to provider finances. There is no publication on efficiency of spending funds. Number of publications on quality is also limited. This can be explained by the fact that they are implemented on an aggregate level, with maybe lack of data sufficient to provide efficiency or quality analysis.

To sum up, this mechanism-endpoint map shows that literature more frequently links a mechanism with a specific endpoint rather than performing a more comprehensive analysis of what types of incentives each financing model creates.

## Conclusions

The review shows which endpoint categories are most frequently examined in association with each payment-mechanism category, but it does not determine whether the mechanisms improve or worsen hospital behaviour. Therefore, the findings should be interpreted as an evidence map rather than a causal synthesis. Evidence is concentrated in endpoint categories related to efficiency of spending funds, quality, access, and hospital finances. Payer spending and budget-based mechanisms are comparatively less represented. These gaps constitute a targeted research agenda, informing priorities for subsequent systematic review and for a database that extracts the direction, magnitude, and context of reported effects.

### Future directions and plans

Future directions for this study include developing a detailed classification of incentive types and extracting the direction, magnitude, and statistical significance of reported findings where available. This will allow subsequent analyses to examine whether specific incentives are associated with consistent patterns across payment mechanisms and healthcare systems. As part of future work, we plan to create a comprehensive database of financial incentives, categorizing them by payment mechanisms, clinical areas, endpoint categories, and reported findings to facilitate further research and policy development. The longer-term goal is to provide actionable evidence for policymakers seeking to optimize financial incentives in ways that support provider performance and patient outcomes.

## Supplementary Information

Below is the link to the electronic supplementary material.

Supplementary Material 1

## Data Availability

The datasets generated and/or analyzed during the current study are not publicly available as they are part of an ongoing study and will be made available after further analysis or the publication of additional findings but can be obtained from the corresponding author upon reasonable request.

## Abbreviations

DRG: Diagnosed–Related–Group
LOS: Length of stay
P4P: Pay for performance
FFS: Fee for service
