# Supplementary material for: Analyzing hospital behavior across different payment mechanism as a response to financial incentives – a scoping review
Source: BMC Health Serv Res. 2026 Jul 2;26:1300. doi: 10.1186/s12913-026-15029-5 (PMC13599200; doi:10.1186/s12913-026-15029-5)
Supplement: Supplementary file 1 [file 12913_2026_15029_MOESM1_ESM.docx]

**Annex 1 - Keyword Selection and Database-Specific Keywords**

PubMed

| **Search** | **Query** | **Results** | **Time** |
| --- | --- | --- | --- |
| #8 | **Search:** ("Hospitals"[Mesh] OR "Inpatients"[Mesh] OR "Beds"[Mesh] OR hospital*[Title/Abstract] OR inpatient*[Title/Abstract] OR beds[Title/Abstract] OR bed[Title/Abstract]) AND ("Medicare Payment Advisory Commission"[Mesh] OR "Health Expenditures"[Mesh] OR "Insurance, Health, Reimbursement"[Mesh] OR "Healthcare Financing"[Mesh] OR "Financing, Organized"[Mesh] OR "Financing, Government"[Mesh] OR "Financing, Construction"[Mesh] OR "Economics"[Mesh] OR "Reimbursement, Incentive"[Mesh] OR "Reimbursement Mechanisms"[Mesh] OR "Insurance, Health, Reimbursement"[Mesh] OR "Outliers, DRG"[Mesh] OR "Insurance Coverage"[Mesh] OR "Universal Health Insurance"[Mesh] OR "Deductibles and Coinsurance"[Mesh] OR "Costs and Cost Analysis"[Mesh] OR Payment*[Title/Abstract] OR financing*[Title/Abstract] OR Reimbursement*[Title/Abstract] OR basket*[Title/Abstract] OR coverage[Title/Abstract] OR co-payment*[Title/Abstract] OR copayment*[Title/Abstract] OR Deductibles[Title/Abstract] OR pricing[Title/Abstract] OR tariff*[Title/Abstract] OR minimal requirement*[Title/Abstract]) AND ("Motivation"[Mesh] OR "Reimbursement, Incentive"[Mesh] OR "Precipitating Factors"[Mesh] OR "Motivation"[Mesh] OR "Quality Indicators, Health Care"[Mesh] OR "Health Status Indicators"[Mesh] OR incentive*[Title/Abstract] OR trigger*[Title/Abstract] OR accelerator*[Title/Abstract] OR activator*[Title/Abstract] OR motivation*[Title/Abstract] OR motivator*[Title/Abstract] OR stimulant*[Title/Abstract] OR deterrent*[Title/Abstract] OR disincentive*[Title/Abstract] OR indicator*[Title/Abstract] OR factor*[Title/Abstract] OR influence*[Title/Abstract] OR behavior*[Title/Abstract] OR behaviour*[Title/Abstract]) **Filters:** in the last 10 years | [19,891](https://pubmed.ncbi.nlm.nih.gov/?term=longquery112f0565fbabc490ed26&filter=datesearch.y_10&ac=no&sort=relevance) | 04:53:17 |
| #7 | **Search:** ("Hospitals"[Mesh] OR "Inpatients"[Mesh] OR "Beds"[Mesh] OR hospital*[Title/Abstract] OR inpatient*[Title/Abstract] OR beds[Title/Abstract] OR bed[Title/Abstract]) AND ("Medicare Payment Advisory Commission"[Mesh] OR "Health Expenditures"[Mesh] OR "Insurance, Health, Reimbursement"[Mesh] OR "Healthcare Financing"[Mesh] OR "Financing, Organized"[Mesh] OR "Financing, Government"[Mesh] OR "Financing, Construction"[Mesh] OR "Economics"[Mesh] OR "Reimbursement Mechanisms"[Mesh] OR "Outliers, DRG"[Mesh] OR "Insurance Coverage"[Mesh] OR "Universal Health Insurance"[Mesh] OR "Deductibles and Coinsurance"[Mesh] OR "Costs and Cost Analysis"[Mesh] OR Payment*[Title/Abstract] OR financing*[Title/Abstract] OR Reimbursement*[Title/Abstract] OR basket*[Title/Abstract] OR coverage[Title/Abstract] OR co-payment*[Title/Abstract] OR copayment*[Title/Abstract] OR Deductibles[Title/Abstract] OR pricing[Title/Abstract] OR tariff*[Title/Abstract] OR minimal requirement*[Title/Abstract]) AND ("Motivation"[Mesh] OR "Reimbursement, Incentive"[Mesh] OR "Precipitating Factors"[Mesh] OR "Quality Indicators, Health Care"[Mesh] OR "Health Status Indicators"[Mesh] OR incentive*[Title/Abstract] OR trigger*[Title/Abstract] OR accelerator*[Title/Abstract] OR activator*[Title/Abstract] OR motivation*[Title/Abstract] OR motivator*[Title/Abstract] OR stimulant*[Title/Abstract] OR deterrent*[Title/Abstract] OR disincentive*[Title/Abstract] OR indicator*[Title/Abstract] OR factor*[Title/Abstract] OR influence*[Title/Abstract] OR behavior*[Title/Abstract] OR behaviour*[Title/Abstract]) | [41,188](https://pubmed.ncbi.nlm.nih.gov/?term=%28%22Hospitals%22%5BMesh%5D+OR+%22Inpatients%22%5BMesh%5D+OR+%22Beds%22%5BMesh%5D+OR+hospital%2A%5BTitle%2FAbstract%5D+OR+inpatient%2A%5BTitle%2FAbstract%5D+OR+beds%5BTitle%2FAbstract%5D+OR+bed%5BTitle%2FAbstract%5D%29+AND+%28%22Medicare+Payment+Advisory+Commission%22%5BMesh%5D+OR+%22Health+Expenditures%22%5BMesh%5D+OR+%22Insurance%2C+Health%2C+Reimbursement%22%5BMesh%5D+OR+%22Healthcare+Financing%22%5BMesh%5D+OR+%22Financing%2C+Organized%22%5BMesh%5D+OR+%22Financing%2C+Government%22%5BMesh%5D+OR+%22Financing%2C+Construction%22%5BMesh%5D+OR+%22Economics%22%5BMesh%5D+OR+%22Reimbursement%2C+Incentive%22%5BMesh%5D+OR+%22Reimbursement+Mechanisms%22%5BMesh%5D+OR+%22Insurance%2C+Health%2C+Reimbursement%22%5BMesh%5D+OR+%22Outliers%2C+DRG%22%5BMesh%5D+OR+%22Insurance+Coverage%22%5BMesh%5D+OR+%22Universal+Health+Insurance%22%5BMesh%5D+OR+%22Deductibles+and+Coinsurance%22%5BMesh%5D+OR+%22Costs+and+Cost+Analysis%22%5BMesh%5D+OR+Payment%2A%5BTitle%2FAbstract%5D+OR+financing%2A%5BTitle%2FAbstract%5D+OR+Reimbursement%2A%5BTitle%2FAbstract%5D+OR+basket%2A%5BTitle%2FAbstract%5D+OR+coverage%5BTitle%2FAbstract%5D+OR+co-payment%2A%5BTitle%2FAbstract%5D+OR+copayment%2A%5BTitle%2FAbstract%5D+OR+Deductibles%5BTitle%2FAbstract%5D+OR+pricing%5BTitle%2FAbstract%5D+OR+tariff%2A%5BTitle%2FAbstract%5D+OR+minimal+requirement%2A%5BTitle%2FAbstract%5D%29+AND+%28%22Motivation%22%5BMesh%5D+OR+%22Reimbursement%2C+Incentive%22%5BMesh%5D+OR+%22Precipitating+Factors%22%5BMesh%5D+OR+%22Motivation%22%5BMesh%5D+OR+%22Quality+Indicators%2C+Health+Care%22%5BMesh%5D+OR+%22Health+Status+Indicators%22%5BMesh%5D+OR+incentive%2A%5BTitle%2FAbstract%5D+OR+trigger%2A%5BTitle%2FAbstract%5D+OR+accelerator%2A%5BTitle%2FAbstract%5D+OR+activator%2A%5BTitle%2FAbstract%5D+OR+motivation%2A%5BTitle%2FAbstract%5D+OR+motivator%2A%5BTitle%2FAbstract%5D+OR+stimulant%2A%5BTitle%2FAbstract%5D+OR+deterrent%2A%5BTitle%2FAbstract%5D+OR+disincentive%2A%5BTitle%2FAbstract%5D+OR+indicator%2A%5BTitle%2FAbstract%5D+OR+factor%2A%5BTitle%2FAbstract%5D+OR+influence%2A%5BTitle%2FAbstra) | 04:53:11 |
| #6 | **Search:** incentive*[Title/Abstract] OR trigger*[Title/Abstract] OR accelerator*[Title/Abstract] OR activator*[Title/Abstract] OR motivation*[Title/Abstract] OR motivator*[Title/Abstract] OR stimulant*[Title/Abstract] OR deterrent*[Title/Abstract] OR disincentive*[Title/Abstract] OR indicator*[Title/Abstract] OR factor*[Title/Abstract] OR influence*[Title/Abstract] OR behavior*[Title/Abstract] OR behaviour*[Title/Abstract] | [7,049,322](https://pubmed.ncbi.nlm.nih.gov/?term=incentive%2A%5BTitle%2FAbstract%5D+OR+trigger%2A%5BTitle%2FAbstract%5D+OR+accelerator%2A%5BTitle%2FAbstract%5D+OR+activator%2A%5BTitle%2FAbstract%5D+OR+motivation%2A%5BTitle%2FAbstract%5D+OR+motivator%2A%5BTitle%2FAbstract%5D+OR+stimulant%2A%5BTitle%2FAbstract%5D+OR+deterrent%2A%5BTitle%2FAbstract%5D+OR+disincentive%2A%5BTitle%2FAbstract%5D+OR+indicator%2A%5BTitle%2FAbstract%5D+OR+factor%2A%5BTitle%2FAbstract%5D+OR+influence%2A%5BTitle%2FAbstract%5D+OR+behavior%2A%5BTitle%2FAbstract%5D+OR+behaviour%2A%5BTitle%2FAbstract%5D&ac=no&sort=relevance) | 04:50:22 |
| #5 | **Search:** "Motivation"[Mesh] OR "Reimbursement, Incentive"[Mesh] OR "Precipitating Factors"[Mesh] OR "Quality Indicators, Health Care"[Mesh] OR "Health Status Indicators"[Mesh] | [557,644](https://pubmed.ncbi.nlm.nih.gov/?term=%22Motivation%22%5BMesh%5D+OR+%22Reimbursement%2C+Incentive%22%5BMesh%5D+OR+%22Precipitating+Factors%22%5BMesh%5D+OR+%22Motivation%22%5BMesh%5D+OR+%22Quality+Indicators%2C+Health+Care%22%5BMesh%5D+OR+%22Health+Status+Indicators%22%5BMesh%5D&ac=no&sort=relevance) | 04:50:07 |
| #4 | **Search:** Payment*[Title/Abstract] OR financing*[Title/Abstract] OR Reimbursement*[Title/Abstract] OR basket*[Title/Abstract] OR coverage[Title/Abstract] OR co-payment*[Title/Abstract] OR copayment*[Title/Abstract] OR Deductibles[Title/Abstract] OR pricing[Title/Abstract] OR tariff*[Title/Abstract] OR minimal requirement*[Title/Abstract] | [238,181](https://pubmed.ncbi.nlm.nih.gov/?term=Payment%2A%5BTitle%2FAbstract%5D+OR+financing%2A%5BTitle%2FAbstract%5D+OR+Reimbursement%2A%5BTitle%2FAbstract%5D+OR+basket%2A%5BTitle%2FAbstract%5D+OR+coverage%5BTitle%2FAbstract%5D+OR+co-payment%2A%5BTitle%2FAbstract%5D+OR+copayment%2A%5BTitle%2FAbstract%5D+OR+Deductibles%5BTitle%2FAbstract%5D+OR+pricing%5BTitle%2FAbstract%5D+OR+tariff%2A%5BTitle%2FAbstract%5D+OR+minimal+requirement%2A%5BTitle%2FAbstract%5D&ac=no&sort=relevance) | 04:49:27 |
| #3 | **Search:** "Medicare Payment Advisory Commission"[Mesh] OR "Health Expenditures"[Mesh] OR "Insurance, Health, Reimbursement"[Mesh] OR "Healthcare Financing"[Mesh] OR "Financing, Organized"[Mesh] OR "Financing, Government"[Mesh] OR "Financing, Construction"[Mesh] OR "Economics"[Mesh] OR "Reimbursement Mechanisms"[Mesh] OR "Outliers, DRG"[Mesh] OR "Insurance Coverage"[Mesh] OR "Universal Health Insurance"[Mesh] OR "Deductibles and Coinsurance"[Mesh] OR "Costs and Cost Analysis"[Mesh] | [654,982](https://pubmed.ncbi.nlm.nih.gov/?term=%22Medicare+Payment+Advisory+Commission%22%5BMesh%5D+OR+%22Health+Expenditures%22%5BMesh%5D+OR+%22Insurance%2C+Health%2C+Reimbursement%22%5BMesh%5D+OR+%22Healthcare+Financing%22%5BMesh%5D+OR+%22Financing%2C+Organized%22%5BMesh%5D+OR+%22Financing%2C+Government%22%5BMesh%5D+OR+%22Financing%2C+Construction%22%5BMesh%5D+OR+%22Economics%22%5BMesh%5D+OR+%22Reimbursement%2C+Incentive%22%5BMesh%5D+OR+%22Reimbursement+Mechanisms%22%5BMesh%5D+OR+%22Insurance%2C+Health%2C+Reimbursement%22%5BMesh%5D+OR+%22Outliers%2C+DRG%22%5BMesh%5D+OR+%22Insurance+Coverage%22%5BMesh%5D+OR+%22Universal+Health+Insurance%22%5BMesh%5D+OR+%22Deductibles+and+Coinsurance%22%5BMesh%5D+OR+%22Costs+and+Cost+Analysis%22%5BMesh%5D&ac=no&sort=relevance) | 04:48:28 |
| #2 | **Search:** hospital*[Title/Abstract] OR inpatient*[Title/Abstract] OR beds[Title/Abstract] OR bed[Title/Abstract] | [1,735,415](https://pubmed.ncbi.nlm.nih.gov/?term=hospital%2A%5BTitle%2FAbstract%5D+OR+inpatient%2A%5BTitle%2FAbstract%5D+OR+beds%5BTitle%2FAbstract%5D+OR+bed%5BTitle%2FAbstract%5D&ac=no&sort=relevance) | 04:48:08 |
| #1 | **Search:** "Hospitals"[Mesh] OR "Inpatients"[Mesh] OR "Beds"[Mesh] | [338,991](https://pubmed.ncbi.nlm.nih.gov/?term=%22Hospitals%22%5BMesh%5D+OR+%22Inpatients%22%5BMesh%5D+OR+%22Beds%22%5BMesh%5D&ac=no&sort=relevance) | 04:47:48 |

Cochrane

| **ID** | **Search** | **Hits** |
| --- | --- | --- |
| #1 | MeSH descriptor: [Hospitals] explode all trees | 4070 |
| #2 | MeSH descriptor: [Inpatients] explode all trees | 1114 |
| #3 | MeSH descriptor: [Beds] explode all trees | 314 |
| #4 | (hospital* OR inpatient* OR beds* OR bed*):ti,ab,kw | 242488 |
| #5 | MeSH descriptor: [Prospective Payment System] explode all trees | 67 |
| #6 | MeSH descriptor: [Health Expenditures] explode all trees | 265 |
| #7 | MeSH descriptor: [Insurance, Health, Reimbursement] explode all trees | 336 |
| #8 | MeSH descriptor: [Financing, Organized] explode all trees | 2097 |
| #9 | MeSH descriptor: [Financing, Government] explode all trees | 857 |
| #10 | MeSH descriptor: [Economics] explode all trees | 13880 |
| #11 | MeSH descriptor: [Healthcare Financing] explode all trees | 8 |
| #12 | MeSH descriptor: [Reimbursement Mechanisms] explode all trees | 301 |
| #13 | MeSH descriptor: [Diagnosis-Related Groups] explode all trees | 63 |
| #14 | MeSH descriptor: [Insurance Coverage] explode all trees | 87 |
| #15 | MeSH descriptor: [Deductibles and Coinsurance] explode all trees | 22 |
| #16 | MeSH descriptor: [Costs and Cost Analysis] explode all trees | 11613 |
| #17 | (Payment* OR financing* OR Reimbursement* OR basket* OR coverage OR co-payment* OR copayment* OR Deductibles OR pricing OR tariff* OR minimal requirement*):ti,ab,kw | 12962 |
| #18 | MeSH descriptor: [Motivation] explode all trees | 9458 |
| #19 | MeSH descriptor: [Reimbursement, Incentive] explode all trees | 143 |
| #20 | MeSH descriptor: [Precipitating Factors] explode all trees | 10 |
| #21 | MeSH descriptor: [Quality Indicators, Health Care] explode all trees | 666 |
| #22 | MeSH descriptor: [Health Status Indicators] explode all trees | 23536 |
| #23 | (incentive* OR trigger* OR accelerator* OR activator* OR motivation* OR motivator* OR stimulant* OR deterrent* OR disincentive* OR indicator* OR factor* OR influence* OR behavior* OR behaviour*):ti,ab,kw | 501700 |
| #24 | (#1 OR #2 OR #3 OR #4) | 242746 |
| #25 | (#5 OR #6 OR #7 OR #8 OR #9 OR #10 OR #11 OR #12 OR #13 OR #14 OR #15 OR #16 OR #17) | 25794 |
| #26 | (#18 OR #19 #20 OR #21 #22 OR #23) | 503328 |
| #27 | #24 AND #25 AND #26 with Cochrane Library publication date Between Jan 2013 and Jan 2023 | 1795 |
